# Editorial for the Special Issue on Flexible Micromanipulators and Micromanipulation

**DOI:** 10.3390/mi14030597

**Published:** 2023-03-04

**Authors:** Alessandro Cammarata

**Affiliations:** Department of Civil Engineering and Architecture (DICAR), University of Catania, 95125 Catania, Italy; alessandro.cammarata@unict.it

The field of micromanipulation is rapidly growing and evolving thanks to advancements in microfabrication technologies and the increased demand for precise and accurate manipulation of microscale objects. The Special Issue on “Flexible Micromanipulators and Micromanipulation” in the *Micromachines* journal provides a platform for researchers to share their latest developments, ideas, and results in this field. This issue includes 12 papers that address various challenges and opportunities in the design, fabrication, and control of flexible micromanipulators. The papers in this Special Issue highlight the latest developments in the field and demonstrate the potential of flexible micromanipulators in addressing some of the most challenging problems in science and engineering.

Kazemzadeh Heris et al. [1] present the design and fabrication of a magnetic actuator for torque and force control using an artificial neural network (ANN)/simulated annealing (SA) algorithm. Kumar et al. [2] propose an electromagnetic micromanipulator levitation system for metal additive manufacturing applications. These two papers demonstrate the potential of magnetic and electromagnetic actuation for micromanipulation and their application in various fields, including additive manufacturing. Xie et al. [3] introduce a novel triaxial parallel compliant manipulator inspired by the tripteron mechanism. This design enables high precision and a large workspace for micromanipulation tasks, which can benefit various fields, including microassembly, microsurgery, and microscale material characterization. Kilikevicius et al. [4] present omnidirectional manipulation of microparticles on a platform subjected to circular motion using dynamic dry friction control. This work provides a promising approach for manipulating microparticles with high precision and speed, which is important in various applications, including biomedical, microelectronics, and environmental fields. Zhang et al. [5] present a micromanipulation and automatic data analysis method to determine the mechanical strength of microparticles. This work provides a fast and accurate method for characterizing the mechanical properties of microscale objects, which can benefit various fields, including material science, biology, and chemistry. Cammarata et al. [6] present a dynamic model of a conjugate-surface flexure hinge, which considers the impacts between cylinders. This work provides an improved understanding of the dynamics of flexure hinges, which is important for the design and control of micromanipulators. Ito et al. [7] propose a vision feedback control for the automation of the pick-and-place of a capillary force gripper. This work provides a promising approach for automating micromanipulation tasks, which is important for reducing human error and increasing efficiency in various fields, including microassembly and biotechnology. Ren et al. [8] propose an optimal design for a 3-PSS flexible parallel micromanipulator based on kinematic and dynamic characteristics. This work provides a systematic approach for designing micromanipulators with high precision and a large workspace, which is important for various fields, including microsurgery and microscale material characterization. Wu et al. [9] propose a condensed substructure approach for kinetostatic modeling of compliant mechanisms with complex topology. This work provides an efficient and accurate method for modeling the behavior of compliant mechanisms, which is important for the design and control of micromanipulators. Cammarata et al. [10] present a direct kinetostatic analysis of a gripper with curved flexures. This work provides a detailed understanding of the behavior of curved flexures, which is important for the design and control of micromanipulators. Baiocco et al. [11] developed a method to measure the Young’s moduli of microcapsules’ shell materials based on diametric compression between two parallel surfaces and numerical modeling. They found a linear relationship between the moduli of the whole microcapsule and the shell material. Tanabe et al. [12] designed a holonomic inchworm robot that can be precisely controlled using four optical encoders. The robot moves in any direction, making it suitable for tasks such as inspection, assembly, and maintenance in tight spaces.

One of the key themes that emerges from the 12 papers is the importance of precise and accurate control in micromanipulation. Several papers present novel control algorithms that enable real-time feedback and adjustment of the micromanipulator’s position, enabling highly precise manipulation of objects at the microscale. Developing new control algorithms and feedback systems is critical to the continued advancement of flexible micromanipulators and their applications.

Another important theme that emerges from the papers in this Special Issue is the diversity of applications for flexible micromanipulators. While some papers focus on biological and medical applications, others explore using flexible micromanipulators in manufacturing, microengineering, and materials science. The ability to manipulate objects at the microscale has enormous potential for a wide range of applications, and the papers in this Special Issue demonstrate the versatility of flexible micromanipulators.

Finally, the papers in this Special Issue also highlight the importance of collaboration between different fields of science and engineering. The development of flexible micromanipulators requires expertise in a range of areas, including materials science, control theory, and robotics. The papers demonstrate the value of collaboration between researchers in these different fields and the potential for interdisciplinary research to drive advances in micromanipulation.

In conclusion, the papers in this Special Issue demonstrate the exciting developments in flexible micromanipulation and the potential of this technology to revolutionize a range of fields. The continued development of flexible micromanipulators and the exploration of new applications will require collaboration between researchers in various disciplines, and this Special Issue highlights the importance of this collaborative approach. We hope that this Special Issue will inspire further research and development in the field of flexible micromanipulation, and we look forward to seeing the future advances that will emerge from this exciting area of research.

## Data Availability

Some data are available from the authors upon request.
